# Efficient Gene Tree Correction Guided by Genome Evolution

**DOI:** 10.1371/journal.pone.0159559

**Published:** 2016-08-11

**Authors:** Emmanuel Noutahi, Magali Semeria, Manuel Lafond, Jonathan Seguin, Bastien Boussau, Laurent Guéguen, Nadia El-Mabrouk, Eric Tannier

**Affiliations:** 1 Département d’Informatique (DIRO), Université de Montréal, H3C3J7 Montréal, Canada; 2 LBBE, UMR CNRS 5558, Université de Lyon 1, F-69622 Villeurbanne, France; 3 INRIA Grenoble Rhône-Alpes, F-38334 Montbonnot, France; University of Lausanne, SWITZERLAND

## Abstract

**Motivations:**

Gene trees inferred solely from multiple alignments of homologous sequences often contain weakly supported and uncertain branches. Information for their full resolution may lie in the dependency between gene families and their genomic context. Integrative methods, using species tree information in addition to sequence information, often rely on a computationally intensive tree space search which forecloses an application to large genomic databases.

**Results:**

We propose a new method, called ProfileNJ, that takes a gene tree with statistical supports on its branches, and corrects its weakly supported parts by using a combination of information from a species tree and a distance matrix. Its low running time enabled us to use it on the whole Ensembl Compara database, for which we propose an alternative, arguably more plausible set of gene trees. This allowed us to perform a genome-wide analysis of duplication and loss patterns on the history of 63 eukaryote species, and predict ancestral gene content and order for all ancestors along the phylogeny.

**Availability:**

A web interface called RefineTree, including ProfileNJ as well as a other gene tree correction methods, which we also test on the Ensembl gene families, is available at: http://www-ens.iro.umontreal.ca/~adbit/polytomysolver.html. The code of ProfileNJ as well as the set of gene trees corrected by ProfileNJ from Ensembl Compara version 73 families are also made available.

## Introduction

Several gene tree databases from whole genomes are available, including Ensembl Compara [1], Hogenom [2], Phog [3], MetaPHOrs [4], PhylomeDB [5], Panther [6]. However they are known to contain many errors and uncertainties, in particular for unstable families [7]. Their use for accurate ancestral genome inference, orthology detection, or the study of genome dynamics could lead to erroneous results. For example Ensembl Compara trees, when reconciled with a species tree to annotate gene duplications and losses, systematically and unrealistically overestimate the number of genes in ancestral genomes, and lead to erroneous predictions of ancestral chromosome structures [8]. It is a known artifact, and a substantial number of nodes in the Ensembl gene trees are labeled as “dubious” [9].

Reasons for errors in gene trees are numerous. When constructed from multiple sequence alignments of homologous genes, they are dependent on gene annotations, gene family clustering or alignment quality, as well as on the accuracy of the models and algorithms used. But above all, gene sequences often do not contain enough substitutions to resolve all the branches of a phylogeny, or alternatively, too many such that the substitution history is saturated. Therefore *sequence based methods*, computing gene trees from sequence information (e.g. PhyML [10], RAxML [11], MrBayes [12], PhyloBayes [13]), are usually accompanied with measures of statistical support on their branches or *a posteriori* distributions of likely trees.

Another category of methods, designated here as *integrative methods*, use a species tree, in addition to a multiple sequence alignment, to model gene gains and losses inferred from the reconciliation between gene and species trees (e.g. TreeBeST [14], TreeFix [15], NOTUNG [16], PhylDog [8], ALE [17], GSR [18, 19], SPIMAP [20], Giga [21], MowgliNNI [22]). They all report gene trees with better accuracy compared with sequence based methods. But they leave a large space for improvement, both on tree quality and on computing time. In terms of models, they often assume unrealistic loss/retention ratios [23]. In terms of computation strategy, most of them use tree space exploration based on small modifications or local moves on branches (typically NNI, SPR, TBR), usually proposed at random. Moves are accepted or rejected according to hill-climbing, Metropolis-like criteria, or other statistical or empirical arguments. Such exploration methods are computationally intensive and do not scale well as databases grow in size. Consequently, database construction pipelines such as TreeBeST (constructing the Ensembl Compara gene trees [1]) have to adopt compromises, exploring a limited tree space. Improving local exploration can be done by using some *correction* techniques, most of them based on the idea of selecting local moves reducing the cost of reconciliation with a species tree (e.g. [16, 22, 24–33]). But even with such improvements, it remains that most local search strategies have no guarantee, neither on running time, nor on the quality of the solution.

In this paper, we propose a new gene tree correction method, called ProfileNJ, which can be directly used as a fast integrative method, without local search. It is a deterministic approach with a guaranteed time complexity. ProfileNJ takes as input a starting tree with supports on its branches, and outputs a set of rooted binary trees containing all well-supported branches of the starting tree, and minimizing the number of duplications and losses when reconciled with a given species tree. It is based on PolytomySolver, a previous algorithm developed by our group [31] for resolving a non-binary tree in a way minimizing a reconciliation cost. Its theoretical complexity has been shown to outperform previous algorithms for the same problem, namely NOTUNG [16] and Zheng and Zhang’s algorithm [34]. ProfileNJ extends PolytomySolver by integrating Neighbor-Joining (NJ) principles to choose among the numerous optimal solutions. Among all trees with equal reconciliation cost exhibiting the same gene count on branches, a choice is made with NJ principles, based on a distance matrix computed from gene sequences. Other extensions are considered such as allowing different costs for duplications and losses, as well as non rooted trees as input. These extensions turn an algorithmic principle into a workable method suited for constructing trees from biological data.

We compare ProfileNJ with TreeFix [15]. Indeed, among all correction methods, TreeFix adopts the most similar evaluation strategy, by exploring neighboring trees which are statistically equivalent according to the sequences. Moreover, TreeFix is among the best available integrative methods, according to the quality of the output trees and running time. On simulations, both algorithms achieve results of comparable quality, but ProfileNJ is several times faster. We also ran ProfileNJ on the whole set of gene families from the Ensembl database, which is out of reach for competing methods with comparable quality. The trees for the whole database were processed by ProfileNJ in less than 48h sequential time. In the same amount of time, 0.5% of the database was corrected by TreeFix. The trees we propose compare very favorably with the trees stored in Ensembl. We then used the reconstructed trees to study genome evolution across the 63 eukaryotic species from the Ensembl database (release 73). A whole genome analysis of duplication patterns is provided, pointing at certain branches which seem to show acceleration of duplication or loss processes.

ProfileNJ is integrated into a modular interface called RefineTree that contains two other gene tree correction tools using information on extant and ancestral synteny [32, 33]. We could thus evaluate the results of a pipeline taking into account gene sequence, gene content and chromosome structure evolution on the Ensembl database according to several criteria: (1) likelihood ratio based on the Ensembl alignments; (2) ancestral genome sizes based on reconciliation with the species tree; (3) linearity of ancestral chromosomal segments computed with DeCo [35]. We discuss the improvements brought by each type of method and the distance of their output to “true” gene trees, in the light of incomplete lineage sorting and gene conversion.

## Description of ProfileNJ

The basic vocabulary of phylogenetic trees is taken from [36], and the reconciliation method between a rooted binary gene tree *G* and a rooted binary species tree *S* is recalled in the Method section. Just note that in a reconciled gene tree *G*, each node (representing an extant gene if at a leaf or an ancestral gene if at an internal node) is mapped to the node of *S* corresponding to the genome the gene belongs to. Edges of *G* are subdivided, adding extra vertices and pending edges so that the extremities of an edge map either to the same node, or to two extremities of an edge of *S*. An internal node of *G* is a duplication if it maps to the same node of *S* as one of its child. The number of genes in a species *s* ∈ *V*(*S*) induced by a reconciled gene tree *G* is defined as the number of nodes *x* ∈ *V*(*G*) mapped to *s*, such that the parent of *x* does not map to *s*. The reconciliation cost is either the number of duplications and losses, or a linear combination of the two if different weights are given to the two kinds of events. Also note that when two trees *G*_1_ and *G*_2_ have the same genes at their leaves, we can say that a branch of *G*_1_ is present in *G*_2_ if the bipartition of the leaves induced by this branch in *G*_1_ is also induced by a branch of *G*_2_.

ProfileNJ is a gene tree correction algorithm that takes as input a gene tree (rooted or non rooted) *G* for a given gene family with supports on its branches, and improves it according to the available information, taken from a species tree, a distance matrix and a threshold number for statistical support. It can be viewed as a generalization of three different standard algorithms designed for evolutionary studies:

- The Wagner parsimony method applied to the inference of ancestral gene contents from the extant gene contents by minimizing a duplication and loss cost [37];
- The Neighbor-Joining [38] (NJ) method which constructs a tree from a distance matrix *D* between taxa;
- The reconciliation of a gene tree *G* with a species tree *S* [39].

Whereas these three methods do not have much in common *a priori*, they are all bricks of our solution and each of them reduces to some particular case of our problem. ProfileNJ outputs a rooted binary gene tree *G*_*c*_ on the same gene family as the input gene tree *G*, where all branches of *G* with a support above the threshold are present in *G*_*c*_. Among all such trees, ProfileNJ outputs those minimizing a duplication and loss cost when reconciled with the species tree with respect to the NJ criterion.

ProfileNJ is an extension of PolytomySolver, a previous algorithm developed by our group [31]. We first describe the principle of the latter and then describe the additions.

**Fig 1:**
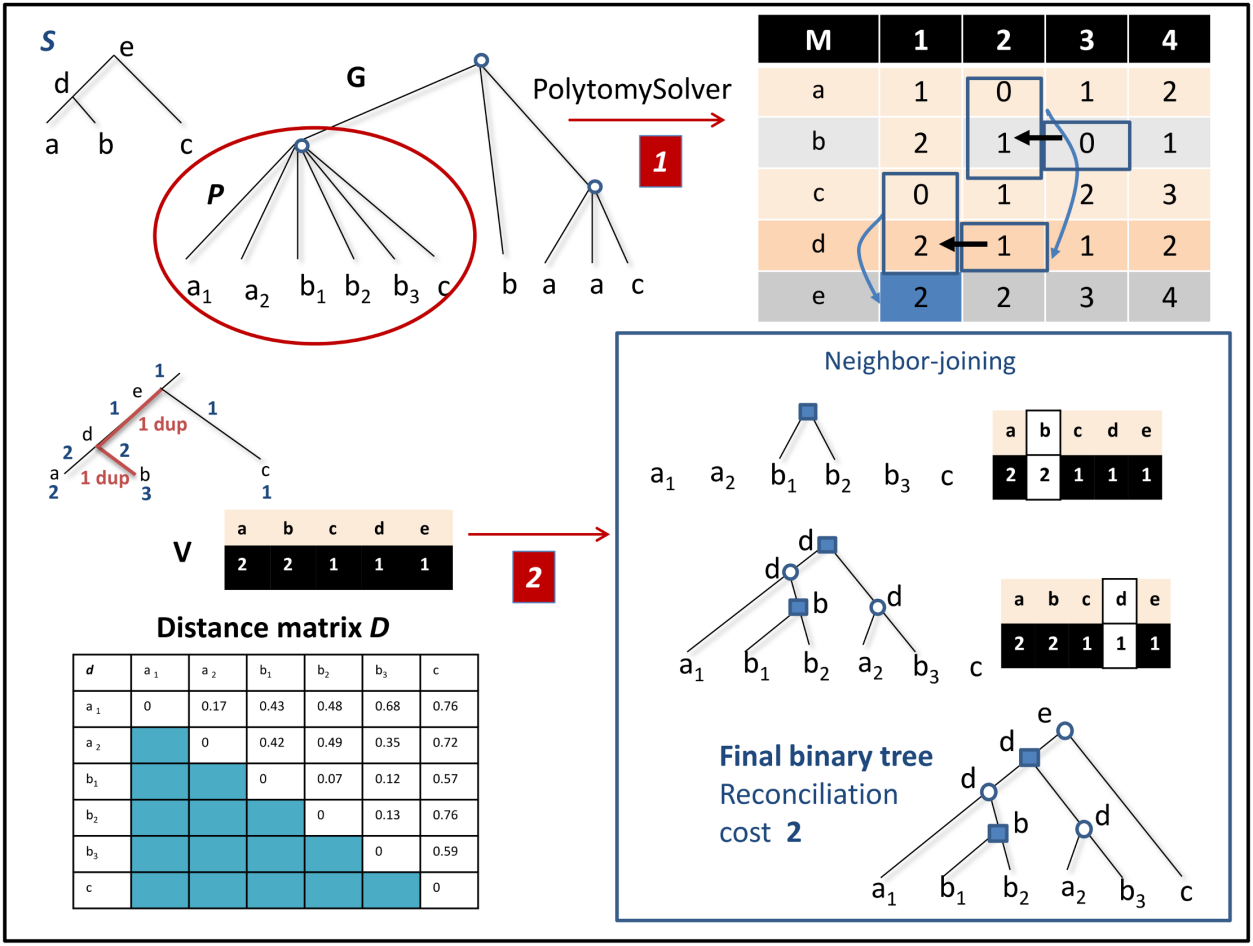
A species tree S and a multifurcated gene tree *G*. Each leaf *x*_*i*_ or *x* of *G* represents a gene belonging to genome *x* present as a leaf in *S*. Step (1) of ProfileNJ is PolytomySolver, which resolves each polytomy *P* of *G* independently. A dynamic programming table *M* is constructed. Step (2) of ProfileNJ takes as input a count vector *V*, here resulting from the backtracking path related by rectangles and arrows in table *M*, and a distance matrix *d* for the considered genes. A Neighbor joining (NJ) based procedure computes the gene tree in agreement with *V* that best reflects the distance matrix. The final completely refined tree is given bottom right. Duplication nodes are indicated by squares.

**PolytomySolver**: It takes as input a multifurcated rooted gene tree *G* (with non-binary nodes) and a binary rooted species tree *S*. It outputs a binary rooted gene tree containing all branches of *G*, that minimizes the number of duplications and losses when reconciled with *S*. It has been shown by [31] that each polytomy (multifurcated node) of *G* can be considered independently. Therefore, in the following, we restrict the presentation to a single polytomy *P* (s.f. polytomy *P* in Fig 1).

The algorithm, based on dynamic programming, computes a table *M* where, for each node (including leaves) *s* of *S* and each integer *k* (limits on *k* are discussed in [31]), *M*(*s*, *k*) is the reconciliation cost of a gene tree with *k* genes in species *s* before any duplication in *s*. For example, in Fig 1, *P* has three genes belonging to genome *b*, and thus *M*(*b*, 1) = 2 as any solution having one gene in *b* before any duplication in *b* means that two duplications must have occurred in *b*, while *M*(*b*, 4) = 1 as having four genes induces one gene loss on *b*.

The final cost of a minimum refinement of the polytomy is given by *M*(*r*, 1), where *r* is the root of *S*. Using a backtracking approach, PolytomySolver then outputs a *count vector* *V* containing the number of genes per node of *S*. Notice that, by construction, two brother nodes of *S* (nodes with the same parent) have the same count. Then a gene tree *G* of minimum cost *M*(*r*, 1) is found, such that in the reconciliation of *G* with *S* there are exactly *V*[*s*] genes in each *s* ∈ *V*(*S*). For example, the final binary tree in Fig 1 has two maximal trees rooted at *b*, as required by the count vector *V*.

If the reconciliation cost is the number of duplications and losses (i.e. the same unit cost is attributed to each duplication or loss), as it was initially published [31], the table *M* can be constructed in time linear in the size of *S* [31], leading to a linear-time algorithm for finding one optimal refinement of the polytomy. Moreover, we showed recently [40] that linearity can be extended to a whole gene tree involving multiple polytomies. For weighted operations, i.e. different costs for duplications and losses, the algorithm runs in quadratic time [40].

**Extensions**: ProfileNJ consists in contracting branches with low support in *G*, which leads to polytomies, and applying Polytomysolver. If *G* is not rooted, one node is chosen as the root. All nodes can be tried and one minimizing the cost can be chosen. The first phases of Polytomysolver are applied, until the construction of a count vector *V*.

Our main extension concerns a treatment of the multiplicity of solutions, as it can be exponential. Indeed, the backtracking procedure mentioned above may lead to many optimal count vectors, and for each count vector, there are possibly several gene trees in agreement with it. Therefore, exploring the set of optimal trees requires exploring the set of all gene trees in agreement with each count vector. For example, the count vector of Fig 1 induces two duplications in *b*. However this vector involves no information on which of the three genes *b*_1_, *b*_2_, *b*_3_ should be joined first. Such information can be deduced from the pairwise alignment distance between gene sequences.

Suppose that a pairwise distance matrix *D* is available for the gene family. Then the problem can be seen as selecting, among all optimal solutions possibly output by PolytomySolver given a vector *V*, the one best reflecting the distance matrix *D*. The problem of constructing a solution such that its induced distance is close to *D* according to a standard measure of metric spaces comparison is NP-complete. But it is also known to be empirically and, to a certain extent, theoretically, well approximated by Neighbor-Joining (NJ) [38, 41]. In ProfileNJ, we use such an NJ approach for choosing neighboring genes.

As in the NJ algorithm, a metric space *E* induced by *D* on the leaves of *P*, is progressively augmented with newly created genes. The algorithm proceeds by successively joining pairs of nodes (points of *E*), eventually leading to a full binary tree. For example, in Fig 1, the initial metric space *E* contains the nodes {*a*_1_, *a*_2_, *b*_1_, *b*_2_, *b*_3_, *c*}. Joining the nodes *b*_1_ and *b*_2_ leads to the new set of nodes {*a*_1_, *a*_2_, *b*_3_, *b*_4_, *c*}. Nodes to be joined are selected according to the NJ criterion, namely we select from a node set of size *n*, the couple of genes *x* and *y* minimizing:

$$\begin{array}{c} Q\left(x,y\right)=\left(n-2\right)D\left(x,y\right)-\sum_{t\neq x}D\left(x,t\right)-\sum_{t\neq y}D\left(y,t\right). \end{array}$$ (1)

The metric space *E* is updated after each join *r* = (*x*, *y*) by removing *x* and *y*, adding a new node *r*, and computing the distance between the newly created node *r* with each element *t* of *E*. When *x* and *y* are not created artificially (i.e. they are not loss nodes created with the last instruction of Algorithm 1), this is done using the NJ formula:

$$\begin{array}{c} D\left(r,t\right)=\frac{1}{2}\left(D\left(x,t\right)+D\left(y,t\right)-D\left(x,y\right)\right) \end{array}$$ (2)

Otherwise, if *x* is a loss, we set *D*(*r*, *t*) = *D*(*y*, *t*) and if *y* is a loss, *D*(*r*, *t*) = *D*(*x*, *t*).

A full pseudo-code of the extension part of ProfileNJ is written as Algorithm 1. It works on one polytomy *P*, assuming that all polytomies below have been resolved. It takes as input a count vector *V*, the species tree *S* and a distance matrix *D* defining the metric space *E*. It outputs a refinement of *P* in agreement with *V*, resulting from the performed joins on the nodes of *E*. Given a node *s* of *S*, denote by *E*(*s*) the subset of *E* restricted to the genes belonging to *s*, and by *m*(*s*) = |*E*(*s*)| the multiplicity of *s* in *E*. The tree *S* is processed bottom-up. For each internal node *s*, speciations are considered first by clustering, using the NJ criterion, the genes from *E*(*s*^*l*^) with the genes from *E*(*s*^*r*^), where *s*^*l*^ and *s*^*r*^ are the two children of *s*. If the obtained multiplicity *m*(*s*) of *s* is greater than the desired count *V*[*s*], then duplications are performed, again using the NJ criterion for choosing the gene pairs in *E*(*s*) to be joined. Otherwise, if *m*(*s*) is lower than the desired count *V*[*s*] of gene copies, then losses are predicted.

**Algorithm 1** ProfileNJ (S,P,V,D)

Let *E* be the metric space with nodes corresponding to the leaves of *P*;

*For* each node *s* of *S* in a bottom-up traversal of *S* *Do*

 *If* *s* is an internal node of *S* with chidren *s*^*l*^, *s*^*r*^ *Do*

  {By construction, *V*[*s*^*l*^] = *V*[*s*^*r*^] = *n*}

  *For* *i* = 1 *to* *n* *Do*

   Choose in *E*(*s*^*l*^) × *E*(*s*^*r*^) the gene pair (*g*^*l*^, *g*^*r*^) minimizing Eq (1) and create

   the node *g* = (*g*^*l*^, *g*^*r*^);

   Remove *g*^*l*^ and *g*^*r*^ from *E* and add *g*;

   Compute *D*(*g*, *g*′) for all *g*′ ∈ *E* using Eq (2);

  *End For*

 *End If*

 *If* *m*(*s*)>*V*[*s*] *Do*

  *For* *i* = 1 *to* *m*(*s*) − *V*[*s*] *Do*

   {Perform *m*(*s*) − *V*[*s*] duplications}

   Choose in *E*(*s*) × *E*(*s*) the gene pair (*g*_1_, *g*_2_) minimizing Eq (1), and create

   the node *g* = (*g*_1_, *g*_2_);

   Remove *g*_1_ and *g*_2_ from *E* and add *g*;

   Compute *D*(*g*, *g*′) for all *g*′ ∈ *E* using Eq (2)

  *End For*

 *End If*

 *Else If* *m*(*s*)<*V*[*s*] *Do*

  {Perform *V*[*s*] − *m*(*s*) losses}

  Add *V*[*s*] − *m*(*s*) artificial genes to *E*(*s*), each with infinite distance to all elements of *E*;

 *End If*

End For

**Complexity**: Let *G* be the solution output by the algorithm, and suppose that *G* has *n* leaves after the inclusion of lost genes. Then exactly *n* − 1 NJ operations have been performed. Each join calculation is restricted to a subset of the genes, and so the time required to perform these joins is bounded by the time required to run the classical NJ algorithm on the *n* leaves of *G*, which is *O*(*n*^3^). Note that *n* can be as large as |*V*(*P*)||*V*(*S*)|, making the worst case running time *O*(|*V*(*P*)|^3^|*V*(*S*)|^3^). However this worst case only occurs when *O*(|*V*(*S*)|) losses are inserted on each branch of the solution. In practice *n* is in *O*(|*V*(*P*)|).

**A Multi-functional algorithm**: ProfileNJ is a phylogenetic tool that generalizes several usually unrelated standard methods. Indeed, if *G* is a binary rooted tree, then ProfileNJ can be seen as a reconciliation tool. If *G* is unrooted, then ProfileNJ can be used to choose an appropriate root according to the induced reconciliation cost. On the other hand, various ways of contracting branches can be considered. For example an exploration scheme contracting the branches one by one and applying ProfileNJ can be considered, which would be equivalent to local modifications [27]. A more radical modification would be to contract all branches, leading to a star tree. In this case, ProfileNJ can be seen as a tool for computing ancestral gene content with Wagner parsimony, minimizing the cost of duplications and losses. If the star tree has all its genes belonging to a single species, ProfileNJ returns an NJ tree. Other kinds of contraction schemes can be imagined, as contracting branches around “Non Apparent Duplications” [42], or “Dubious duplications” stored in the Ensembl trees.

Notice that the pseudo-code for ProfileNJ has been given for a single count vector. For a full exploration of the tree space, all count vectors should be considered. This is what we do in the simulation section.

## Efficiency of ProfileNJ

### Efficiency of the NJ criterion

We ran ProfileNJ twice on the same data set of 20519 trees (the Ensembl Compara gene families), except that once the distance matrix was computed using the Ensembl nucleotide alignments with FastDist from the FastPhylo package [43], and once the distance matrix was random. The starting tree was computed for every family using PhyML on the nucleic alignments, and all branches with aLRT support <0.95 were contracted. In average 55% of the branches were contracted. A histogram of the full distribution is shown in Fig A in S1 File.

Then we computed the likelihood of both trees for every family with PhyML. Among the trees for which the likelihood was different (55% of all tested trees), 76% were in favor of the trees built with the FastDist distance matrix. These 76% of trees account for 95% of the total sum of likelihood differences on all trees.

The comparisons are clearly in favor of the NJ criterion over no criterion at all, while quantitatively there remains a small but non negligible part of the trees for which no criterion (the random distance matrix) gives an unexplained slightly, but significantly, better likelihood.

### Efficiency of the tree space exploration strategy on simulated gene trees

We compared ProfileNJ with TreeFix, the most closely related tool, on simulated data. The principle of TreeFix is to randomly explore, by local moves, the space of trees that are statistically equivalent to the input tree, and report the one with the best reconciliation cost. Instead, we take a deterministic and more targeted approach by focusing on weakly supported branches of the tree, with a possibly deep modification of the tree. The comparison with TreeFix is therefore intended to compare these two space exploration strategies.

In [15], TreeFix has been compared with NOTUNG [16] and SPIMAP [20], showing a better accuracy than NOTUNG, and a higher speed than SPIMAP. We perform a similar comparison on the same simulated dataset of 16 fungi. This dataset consists of simulated gene families generated under the SPIMAP model and their corresponding nucleotide alignments, for four different rates of duplication and loss (DL) events: (1 × *r*_*D*_, 1 × *r*_*L*_), (2 × *r*_*D*_, 2 × *r*_*L*_), (4 × *r*_*D*_, 4 × *r*_*L*_) and (4 × *r*_*D*_, 1 × *r*_*L*_), where *r*_*D*_ and *r*_*L*_ are respectively the estimated duplication and loss rates for fungi. For instance, a (2 × *r*_*D*_, 2 × *r*_*L*_)-simulated gene family is expected to have, on average, two times more duplications and losses than a real gene family in fungi. Comparisons reported in this section are performed on 2575 simulated gene families randomly chosen from the four fungi datasets with different DL rates.

An initial maximum likelihood (ML) tree is constructed for each simulated gene family with RAxML v-8.1.2 [11], with the rapid bootstrap algorithm, under the GTR-Γ model and the majority rule consensus tree as bootstopping criterion. A randomly rooted tree is then provided as input to TreeFix (as TreeFix requires the input tree to be rooted), while a multifurcated non rooted tree obtained by contracting the branches with support lower than 95% is provided as input to ProfileNJ. We used default parameters for both programs. Among the set of all binary trees output by ProfileNJ (one for each count vector), the best statistically supported tree was selected using RAxML under the GTR-Γ model of nucleotide substitution.

For RAxML, TreeFix and ProfileNJ trees, we measured the Robinson-Foulds (RF) distance to true trees, compared the reconstructed tree with the true tree using site-wise likelihoods (see Fig G in S1 File), measured the accuracy of the duplication and loss scenarios (Fig E in S1 File), the sensitivity of the accuracy to gene family size (Fig F in S1 File), the sensitivity to species tree errors (Fig H in S1 File), and the running time.

**Fig 2:**
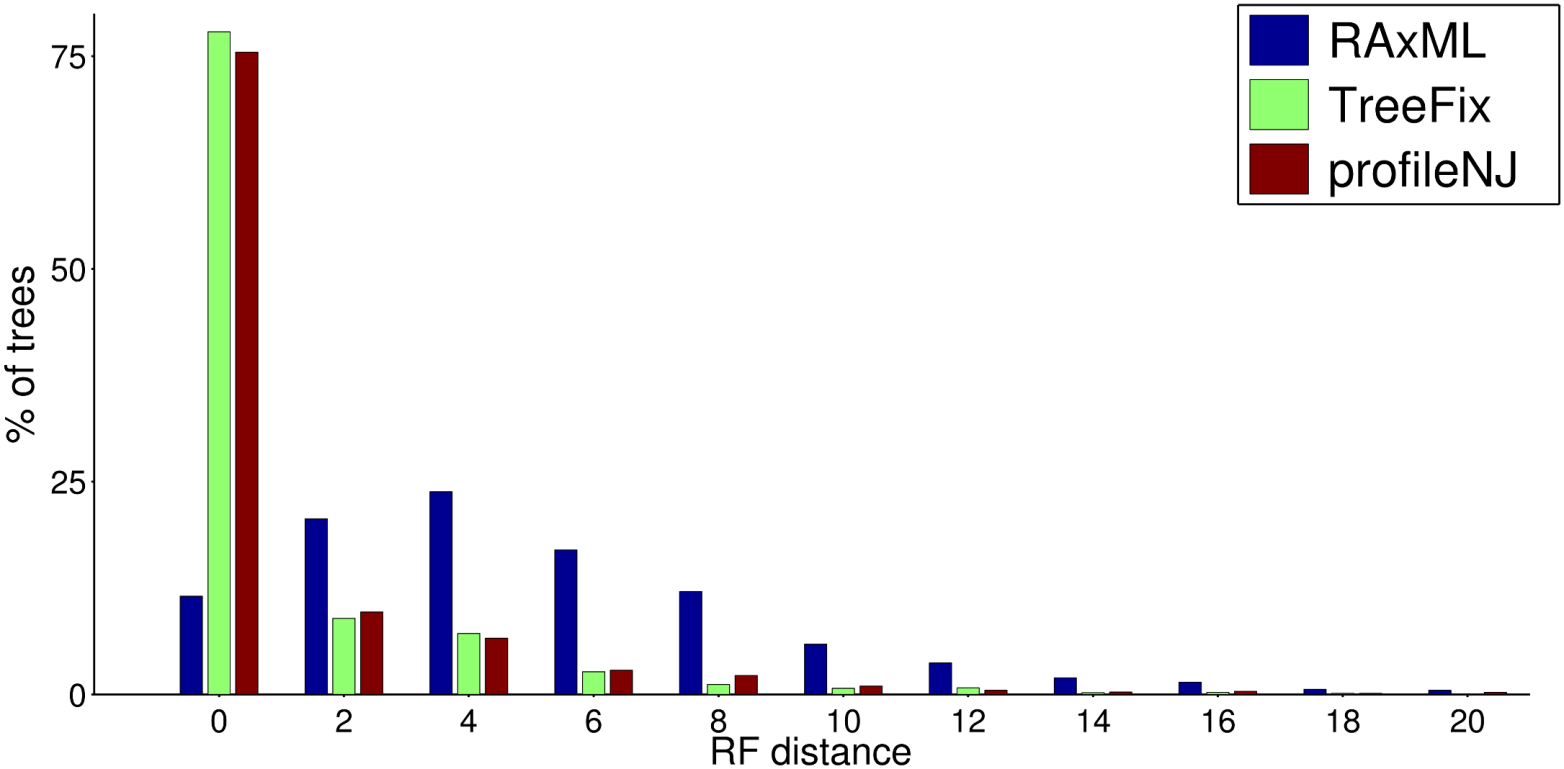
Topology accuracy of RAxML, TreeFix and ProfileNJ trees, measured by RF distance with the true tree, on ∼ 2500 simulated trees from the fungal dataset. We use a sample of trees simulated under four different DL rate: (1*r*_*D*_—1*r*_*L*_), (2*r*_*D*_—2*r*_*L*_), (4*r*_*D*_—4*r*_*L*_) and (4*r*_*D*_—1*r*_*L*_). Percentage of reconstructed trees (y-axis) with a given RF distance (x-axis) to the true tree. TreeFix and ProfileNJ have a similar reconstruction accuracy (75% of trees match the true trees) while the input trees (RAxML) have the lowest accuracy. The graph is cut on the right, but contains more than 99% of the data.

Fig 2 illustrates the results for the RF distance. It shows that sequence-only does not contain enough signal to lead to the true tree for our simulated dataset, and integrating additional information from the species tree actually improves reconstruction. Indeed, TreeFix and ProfileNJ reconstruct around 75% of true trees, compared with only 10% for RAxML. We investigated some cases where erroneous gene trees were inferred, and found that often, the true scenario was not parsimonious in terms of duplications and losses, while TreeFix and ProfileNJ chose duplications that are too recent in order to avoid losses. An example is given in Fig D in S1 File.

The performances of TreeFix and ProfileNJ are similar in terms of distance to the true tree. As for RAxML, it gives the best likelihood, which is not surprising as it is specifically designed for that. The returned likelihood is even usually higher than the likelihood of the true tree, but not significantly according to an AU test (Approximately unbiased [44], see Fig G in S1 File). TreeFix is designed to produce trees which are not significantly different than the ML tree, which we could check: 1.36% of the trees fail the AU test against the ML tree at *α* = 0.05, while the proportion jumps to 9.17% for ProfileNJ. It is noticeable that this has no visible consequence on the distance to the true tree (Fig C in S1 File).

**Fig 3:**
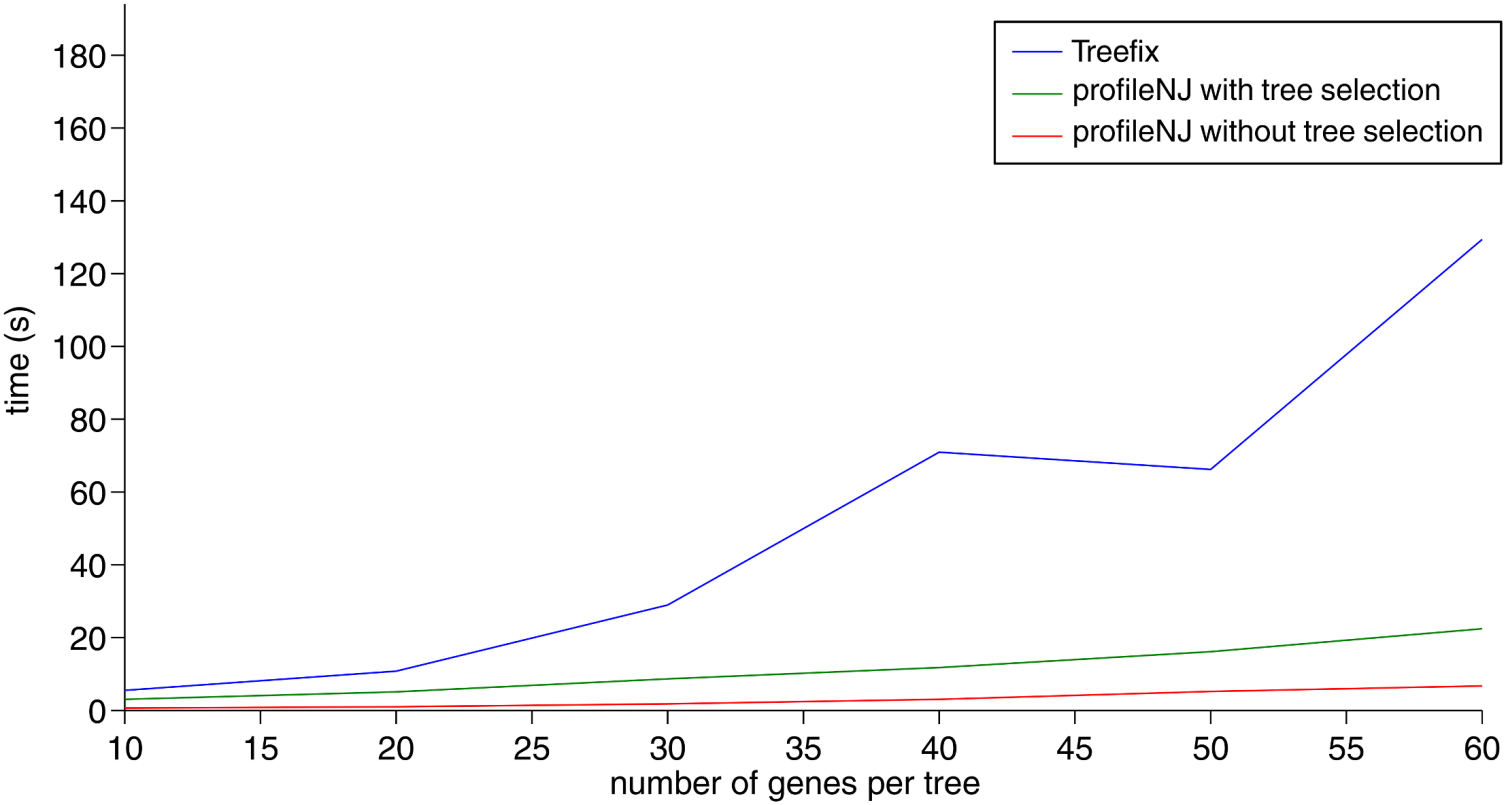
Run time of TreeFix and ProfileNJ for increasing size of gene tree.

Fig 3 shows that ProfileNJ outperforms TreeFix in running-time, the gap between the two algorithms increasing with tree size. This figure also shows that the most time-consuming step in ProfileNJ is tree selection. For a tree of size 30, ProfileNJ is about four to seven times faster than TreeFix, and about 15 times faster if we discard statistical support evaluation and tree selection step with RAxML. This includes the construction of the distance matrix, but not the construction of the initial RAxML, as it is common to both methods.

Other analyses, including the sensitivity to gene family size and the number of duplications and losses, are reported in S1 File. They lead to the same conclusions: TreeFix and ProfileNJ have similar performance on all measures except running time for which ProfileNJ is significantly better.

## Application on biological data

### Software: RefineTree

**Fig 4:**
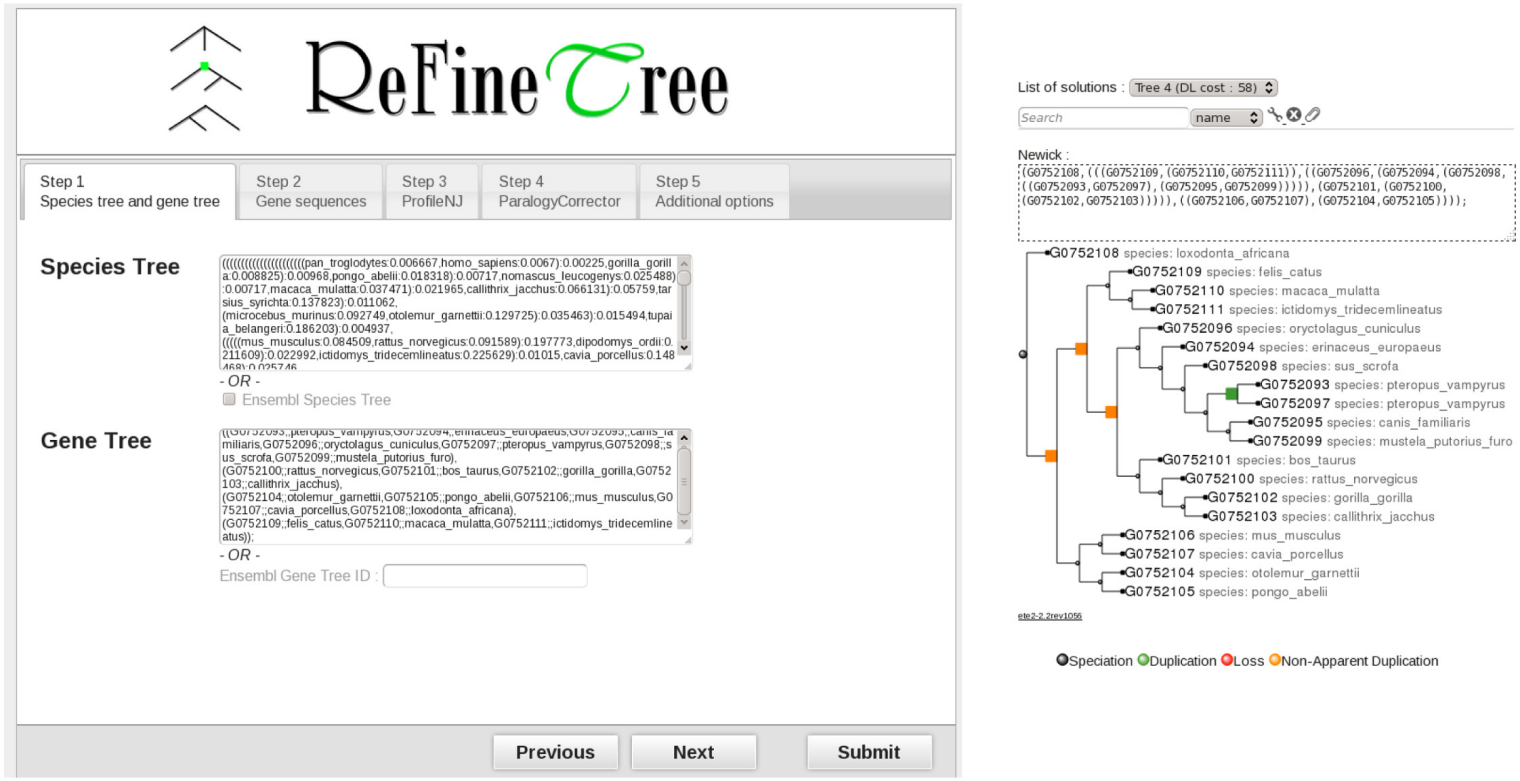
RefineTree web interface. The input is a species tree (or by default the Ensembl species tree) and a gene tree (or an Ensembl gene tree ID), gene sequences and additional options such as the branch contraction threshold, the request to test all roots, the maximum number of trees to be output by ProfileNJ and sorted by likelihood, etc. The integrated algorithms are ProfileNJ and ParalogyCorrector. Using this second algorithm requires, in addition, the input of a set of orthology constraints.

ProfileNJ is integrated in a new modular online software, called RefineTree, designed to combine a number of correction techniques, with an easy-to-use interface (see Fig 4). The present version includes, in addition to ProfileNJ, a tool called ParalogyCorrector [32] for correcting orthology relations. ParalogyCorrector takes as input a gene tree and a set of known pairwise orthology relations between genes, which would typically be derived from synteny comparisons, and constructs the tree which is the closest to the input tree according to the RF distance, with the constraint that couples of putative orthologs must be orthologs in the reconciliation (see Method section).

RefineTree can be used in a modular way, according to the user’s specifications. It has been designed to be easily extensible to other tools. For example instead of asking the user to input his own orthology relations, tools for inferring putative orthologs can be included.

### Protocol

The low running time of ProfileNJ makes it possible to run it on large databases. Here, we exhaustively use ProfileNJ to correct all trees of the Ensembl Compara database, containing 63 eukaryotic whole genomes. Gene trees are constructed for 20519 families. In order to quantify the contribution of ProfileNJ and the contribution of methods using other kinds of information as synteny, we compared three sets of trees on the whole database.

**Fig 5:**
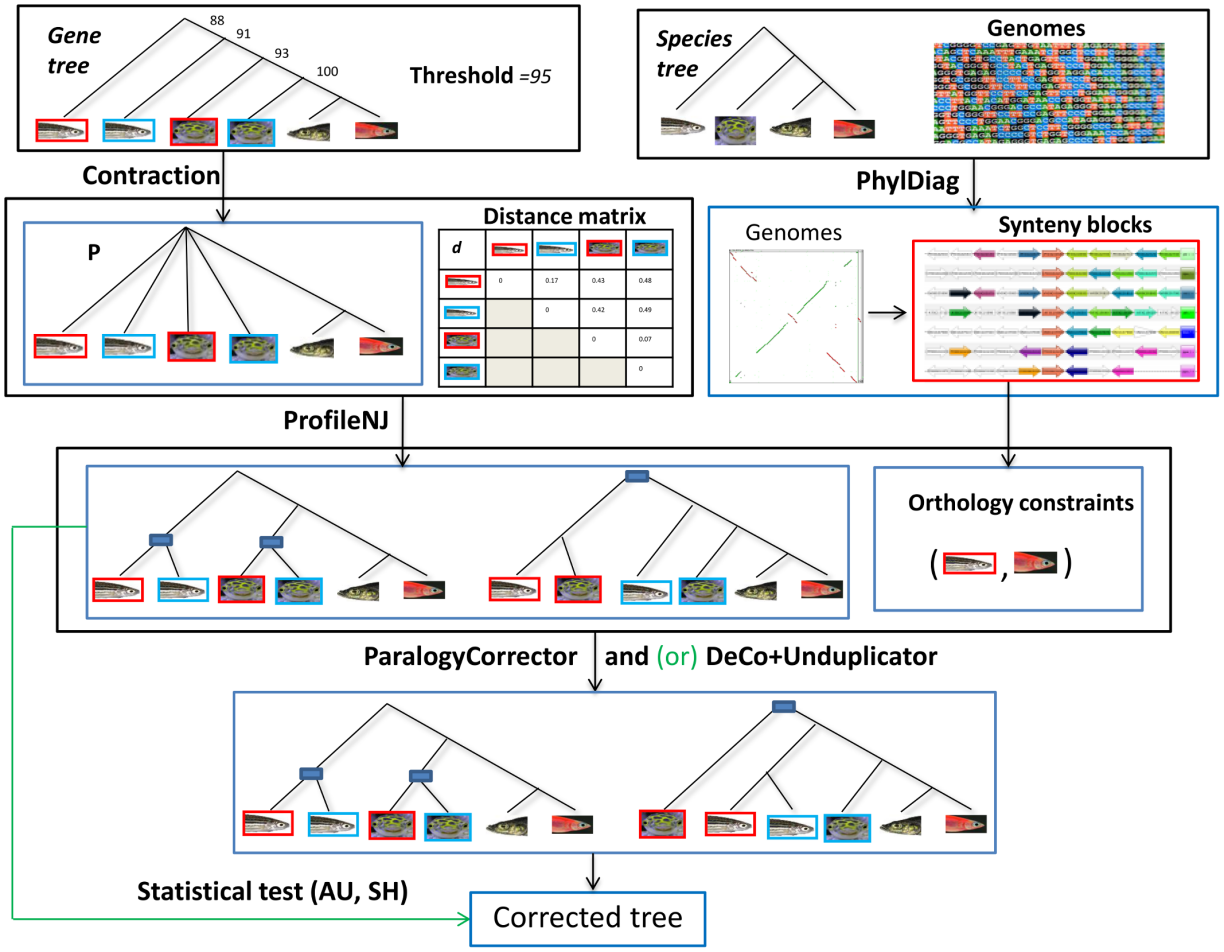
A general view on RefineTree when run on the Ensembl Compara gene families. An example is given for a species tree *S* of four fish species, a gene family of six genes (a gene is represented by the picture of the species it belongs to, and two paralogs belonging to the same species are distinguished by a different frame color), a rooted gene tree *G* (although it can be non rooted in general) with branch support, and a given threshold for branch contraction. Data framed in black are the input and those framed in blue are the output of the correction algorithm labeling the edge linking the considered frames. Black arrows depict the use we make of RefineTree on the Ensembl gene trees. The green arrow and the green “or” are alternative uses avoiding one or both of the correction tools ParalogyCorrector and Unduplicator. Any framed set of data can be alternatively provided to the pipeline as input. For example, orthology constraints obtained from various sources can be directly provided as input to ParalogyCorrector. The method for inferring orthology constraints from synteny blocks is described in the text.

- **Ensembl trees**: Trees stored in the Ensembl gene family database (see Method section);
- **ProfileNJ trees**: Trees output by ProfileNJ with unrooted PhyML trees as input (where branches with aLRT support <0.95 are contracted) and FastDist distance matrices. A single solution is retained for the rooting leading to a minimum weighted reconciliation cost (see Method section);
- **Synteny trees**: Trees output by either ParalogyCorrector or Unduplicator [32] (the two are computed and the most likely according to the sequence is chosen) with ProfileNJ trees as input, using PhylDiag [45] and DeCo [35] to infer synteny constraints (see Method section and Fig 5)

### Results on Ensembl gene trees

The PhyML trees for the 20519 gene families were processed by ProfileNJ in less than 48h sequential time. In the same amount of time, 0.5% of the database was corrected by TreeFix.

We evaluated the resulting trees (all made available at the web page indicated in the abstract) according to sequence likelihood, ancestral genome content and ancestral chromosome linearity. The ancestral genome content metric is based on the assumption that the distribution of ancestral gene content sizes should be close to that of extant genomes. Incorrect trees are known to require additional duplications to be reconciled with the species tree, which tends to increase the number of genes in ancestral genomes. The ancestral chromosome linearity metric is based on the assumption that the linearity of ancestral genomes is expected to be as close as possible to that of the extant genomes, with each gene having zero, one or two neighbors, with a peak at two (having genes with zero or one neighbor is usually due to partially assembled genomes).

**Fig 6:**
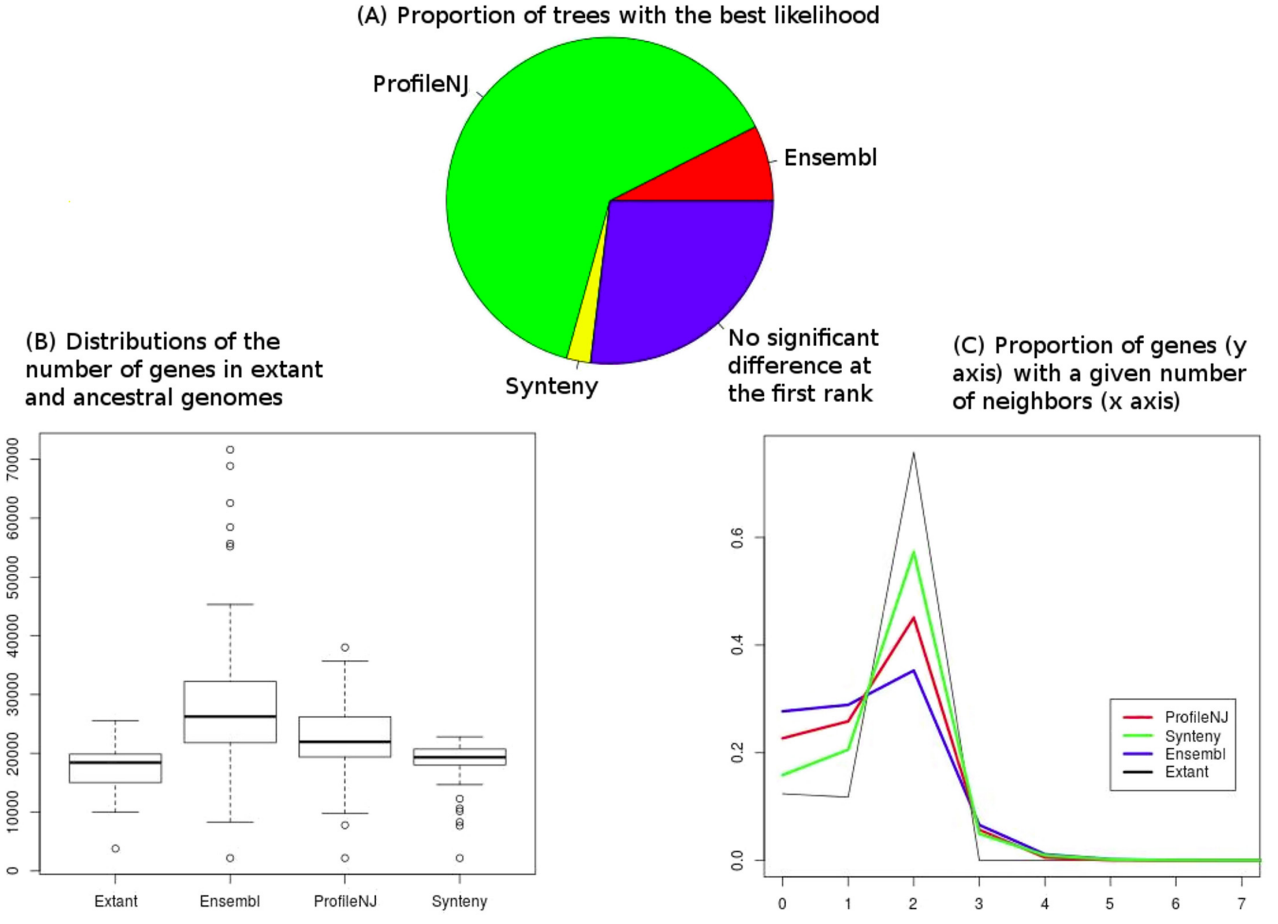
Sequence likelihood, ancestral genome content and ancestral chromosome linearity for ProfileNJ, Synteny and Ensembl trees. **(A)** Proportion of trees with a significantly better likelihood computed with PhyML. AU tests were computed for the three trees for each family, and if the tree at the first rank was significantly better than the second, it was stored as the best likelihood, and if not, it was stored as “no significant difference at the first rank”. **(B)** Gene content computed with DeCo. Gene content has one value for each node of the phylogeny of 63 species, except for extant genomes, for which it has one value for each leaf. **(C)** Genome linearity computed with DeCo. Genome linearity is represented by a graph, whose *x* axis is the number of neighbors a gene can have, and the *y* axis shows the proportion of genes having this number of neighbors. Parameters from extant genomes are given as a reference in (B) and (C). Statistics for ancestral genomes are assumed better when close to the extant ones.

Results are given in Fig 6. ProfileNJ trees show a better behavior than Ensembl trees according to the three measures: more than 2/3 of the trees have a better likelihood than Ensembl trees, ancestral genome content distribution is much closer to the extant one, and linearity of chromosomes is higher. Therefore this set of trees, achieving better performance according to sequence evolution, gene content evolution and chromosome evolution, is arguably a better dataset than the one stored in the Ensembl database.

However, results obtained when we include synteny information are less clear. Indeed, quality of synteny trees drops in terms of likelihood (Fig 6(A)), but jumps in terms of the stability of gene content and the linearity of ancestral chromosomes (Fig 6(B) and 6(C)).

### Results on duplication and loss history in Eukaryotes

Partial patterns of duplications and losses in eukaryotes have been considered in previous studies, as for example by [8] in mammals with a subset of gene families, or by [46] in vertebrates with a subset of species. The ability of ProfileNJ to handle the whole Ensembl database allowed us to perform a more exhaustive study. In addition to gene trees, we reconstructed all ancestral gene contents and organizations. Gene content is computed according to reconciliation (see Methods), and genome organization, which consists in sets of links between consecutive genes, is inferred with DeCo. Genes are not always clustered into full linear genomes. Such non-linearity has diverse causes that we do not wish to mask with an *ad-hoc* linearization method. An interesting property of DeCo is to highlight genes or groups of genes evolving together in parts of the tree. For example 8488 blocks of co-duplicated genes are inferred by DeCo on the considered eukaryote dataset. Most of them contain only a few number of genes (83% contain 2 genes). The largest blocks are found in the terminal branches leading to *Danio rerio* and *Caenorhabditis elegans*.

**Fig 7:**
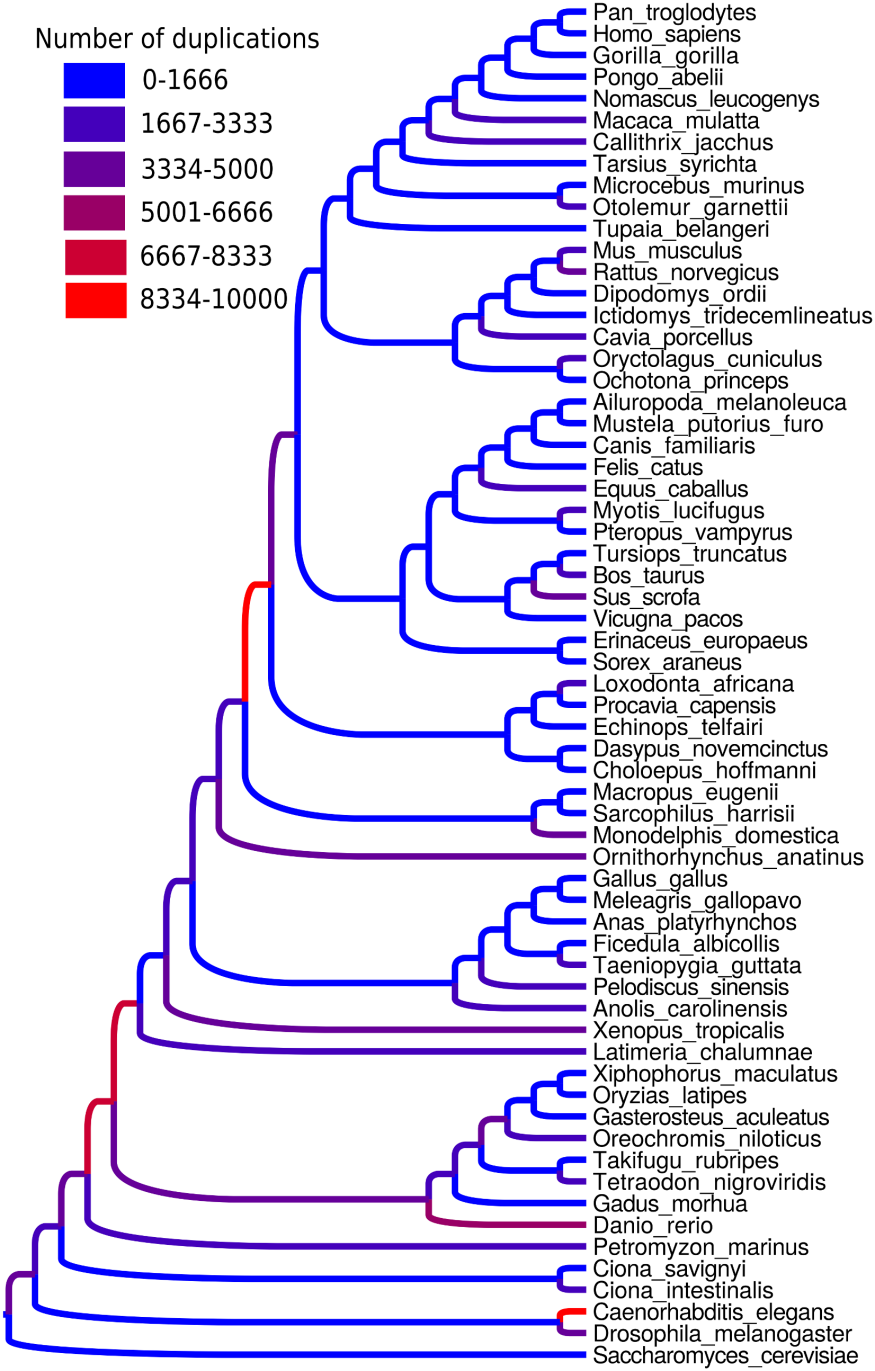
Numbers of duplications in the eukaryote phylogeny, estimated with reconciled ProfileNJ trees from PhyML starting trees on the whole Ensembl Compara database, version 73. Drawn with Figtree [49].

Fig 7 shows the result for the full genomes of the full phylogeny of the 63 Ensembl species. As seen in Fig 7, duplication rates are highly variable across branches of the phylogeny. Branches with a large number of duplications (hot branches) are those leading to vertebrates, which is in agreement with the two rounds of whole genome duplication hypothesis. Interestingly, the speciation event leading to *Petromyzon marinus*, which is usually thought to have diverged after these events [47], precedes the hot branches. This may be in agreement with recent results based on the analysis of Hox clusters in the Japanese lamprey [48]. Another hot branch leads to eutherian mammals, which was also found by two other studies [8, 46] with partial data. These two hottest internal branches are exactly the ones found by Mahmudi et al [46] using a probabilistic technique, but using only 9 species due to computational cost. Other hot branches are terminal, the hottest being those leading to *Caenorhabditis elegans* and *Danio rerio*. This is possibly due to ongoing dynamics of polymorphic copy number variations. The same tree showing the number of losses is provided in Fig I in S1 File.

## Discussion

ProfileNJ is a new gene tree correction method based on exploring a restricted tree space and choosing the most likely tree according to a species tree and a distance matrix on gene sequences. It is shown to be accurate and it outperforms in running time the most comparable existing correction methods. Efficiency in running time allowed us to apply ProfileNJ to the entire Ensembl database.

Trees obtained by correcting PhyML trees with ProfileNJ are arguably better than gene trees stored in Ensembl, according to sequence likelihood, ancestral genome content and ancestral chromosome linearity. We also corrected directly the Ensembl trees and the results (not shown) were similar, ProfileNJ giving better ancestral genomes and more likely trees than the starting trees. Based on such accurate trees, we have been able to perform an exhaustive study of the patterns of duplication and loss on the phylogeny of the 63 Eukaryote species included in Ensembl.

### Accounting for synteny

In addition to sequence and phylogeny, we also used synteny information and orthology relations as correction criteria. As gene trees contain the most complete information about a gene family history, detecting orthologs or studying gene repertoire evolution should be achieved by interpreting trees. But due to the rate of errors in the current trees stored in databases, orthology is often assessed with a series of techniques including synteny [50] and Reciprocal Best Hits, while the evolution of gene repertoires is often studied with phyletic profile techniques [51]. What we presented here is a way of integrating those diverse techniques into a phylogenetic framework.

Unfortunately, integrating synteny information results in a drop of gene tree quality in terms of likelihood. One interpretation is that achieving orthology constraints may require breaking well supported branches. Part of the likelihood drop could also be interpreted as an inadequacy of the sequence evolution models to appropriately account for gene families with a high rate of duplications. Further, as observed in our simulations, the true tree is not necessarily the ML tree. Finally, the likelihood is computed with an alignment that usually results from a guiding tree, estimated using fast but crude approaches, and often different from the tested tree. Some synteny trees might therefore be better trees even in cases where sequence likelihood disagrees, because sequence likelihood can be incorrect.

However there is a third interpretation. Synteny information describes the history of loci [52], while phylogenetic models describe the evolution of sequences. Loci and sequences often have the same history, but they may differ following gene conversion or incomplete lineage sorting (ILS).

**Fig 8:**
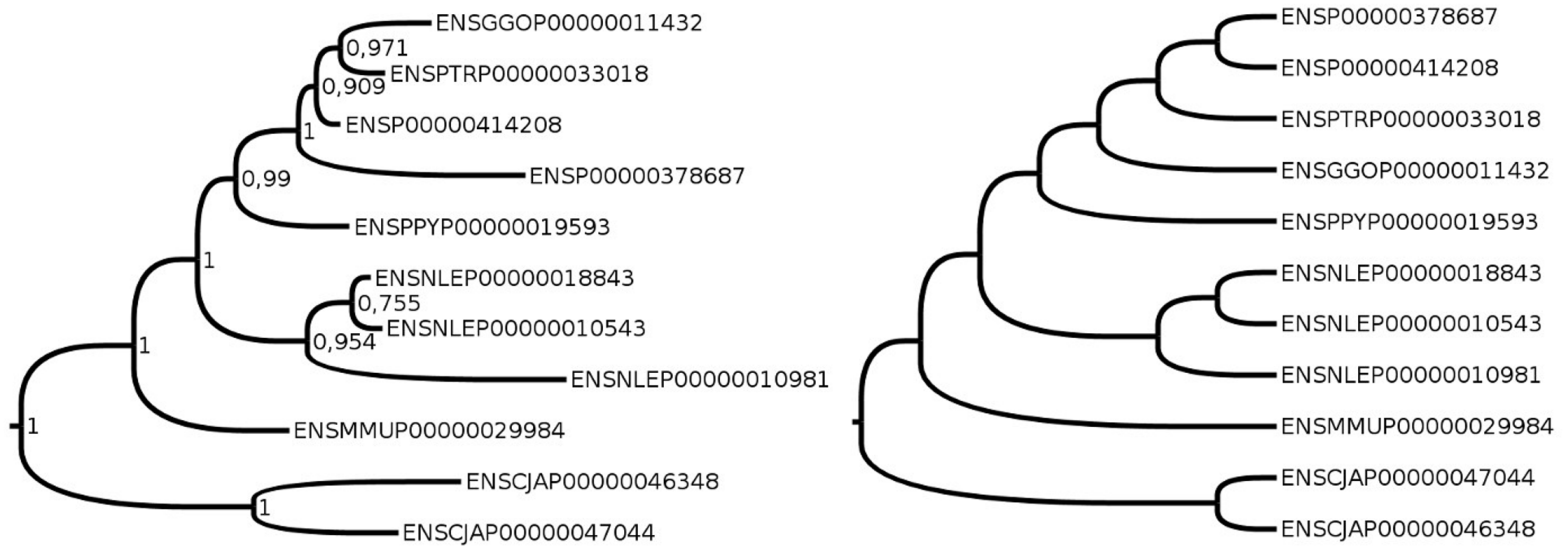
A probable example of ILS visible on a subtree of an Ensembl gene family. The monophyly of the chimpanzee and gorilla genes (ENSPTRP00000033018 and ENSGGOP00000011432) is well supported by the sequences (left tree, constructed by PhyML, with aLRT supports), while synteny argues for orthology of both with the human genes (ENSP00000414208 and ENSP00000378687) (right tree, constructed by ProfileNJ followed by ParalogyCorrector), so that a scenario of duplications and losses compatible with the left tree is unlikely.

In case of ILS or gene conversion, two different true versions of the gene history are concurrent. In Fig 8 the gene as a locus has a history depicted by the right tree, while the gene as a sequence has a history depicted by the left tree. None of the two are wrong, but they are significantly different. They highlight the ambiguity of the definition of a gene, which yields an ambiguity in its history. Sequence trees will have a high likelihood and mediocre results for gene contents and synteny when constructed from duplication and loss scenarios, while it is the opposite for locus trees. A probabilistic model that incorporates ILS in sequence and duplications and losses in loci has been proposed [52]. However, no model is currently able to handle conversion.

### Not only the gene trees

Using genome evolution in the construction of the gene trees, we get ancestral genomes as a byproduct. They are made of genes and sets of gene adjacencies. They are still too big (in terms of gene number) and too non linear to be fully trusted. This is partly due to incorrect gene trees in our output, or incorrect inferences from DeCo, but also to problems in sequencing, assembling, annotating genomes, clustering families or inferring the species tree. Good methods for finding linear structures from a set of adjacencies exist [53]. Here we rather used non-linearity as a testimony of the flaws of the data and methods used to reconstruct genome evolution.

Although gene trees are “better” with our correction, they still could be improved. The likelihood drop for synteny correction is indeed surprising, as these corrections lead to ancestral genomes that are closer to gene content and gene neighborhoods of extant genomes. We would need better exploration schemes with integrated models to really trust gene trees on a whole genome database within a deep phylogeny.

## Methods

Families, alignments and trees were taken from Ensembl Compara release 73, sept 2013. They were computed with a pipeline called TreeBest, but we simply call them the “Ensembl trees”. There are 20529 rooted trees, each corresponding to a gene family, for a total of 1091891 genes taken from 63 species. 13128 of these trees have 3 leaves or more, and are thus treated by our phylogenetic study. Information on gene position on chromosomes, scaffolds or contigs is available at ftp://ftp.ensembl.org/pub/release-73/emf/ensembl-compara/homologies/.

### Use of ProfileNJ on Ensembl

PhyML was used with default parameters to compute maximum likelihood trees from the protein multiple alignments taken from Ensembl, for families with 4 sequences or more. An aLRT support was computed, and only branches with support ≥0.95 were preserved by ProfileNJ. FastDist was run on DNA alignments to provide a distance matrix. Then ProfileNJ was run with the command (an example is given for the first family).

ProfileNJ -s Compara.73.species_tree \\

 -g data/famille_1.start_tree \\

 -d data/famille_1.dist \\

 -o data/famille_1.tree \\

 -n -r best -c nj --slimit 1 \\

 --plimit 1 --firstbest --cost 1 0.99999

We tested the sensitivity of the method to the choice of the threshold parameter for contracting unsupported branches. The threshold is a trade-off between the amount of change in a tree and the probability that the resulting tree is rejected. Values that are too high would avoid exploring a large space around the starting tree while small values would lead to low likelihood trees. It has to be settled empirically. For example.80 bootstrap threshold was considered an acceptable threshold in some genomic studies [54].

### Ancestral genes and genomes with DeCo

DeCo [35] computes ancestral genes and gene neighborhoods from gene trees and extant gene neighborhoods. It was used to compare ancestral gene contents and orders with different sets of gene trees for the same set of gene families to generate Fig 6. It takes as input rooted gene trees and *adjacencies*, *i.e.* couples of extant genes that are consecutive on a chromosomes. Ancestral adjacencies are constructed according to a parsimony principle minimizing the number of gains and losses of adjacencies.

Adjacencies at different loci on chromosomes are supposed to evolve independently one from the other. As a consequence, although an extant gene should belong to at most two adjacencies (zero or one is frequent because of low assembly quality of some genomes), it is not necessarily the case for ancestral genes. As a consequence ancestral genomes are not necessarily linear arrangements of genes. It might be seen as a weakness of this ancestral genome reconstruction method, but for us it is a criterion to evaluate the quality of gene trees. Indeed, a high part of the non linearity of ancestral genomes is not due to the inadequacy of the software itself, but to the quality of the input data. It has been remarked that a significant improvement in the linearity of ancestral genomes was obtained by constructing gene trees according to more integrative models [8, 55].

### Information from extant synteny

Orthology constraints for synteny trees are inferred as follows. If several genes are found consecutive in one genome, and their homologs are also found consecutive in the other genome, the common linear arrangement was in the ancestor and the homologous genes are probably orthologous. This hypothesis is incorrect in at least three cases: (1) if the whole block of genes was duplicated, (2) if there is a tandem duplication of a gene followed by a differential loss in the two species, or (3) if a gene is converted by a paralog. To handle these cases, we require that (1) the majority of the homologous genes are indeed predicted as orthologs by phylogeny, (2) the common ancestor of two homologous genes does not lead to two paralogous descendants placed in tandem in one species. In case (3), we are in a situation where the loci are orthologous but not the sequences. In that case we construct the “locus tree” [52] and trust syntenic information over gene sequence information.

First we ran PhylDiag as follows, for each pair of genomes. Files genome_1, genome_2 and ancestral_genes respectively contain the ordered list of genes from each genome, and the list of families clustering the genes as in the Ensembl database.

phylDiag.py genome_1 genome_2 ancestral_genes \\

 -gapMax = 2 -pThreshold = 0.00000005 \\

 -filterType = InBothSpecies -multiprocess \\

 -minChromLength = 2 >syntenyblocks_1_2

The statistical threshold is calculated in order to minimize the number of false positives, taking into account the number (2211) of comparisons between pairs of species and an expected number (500, as an approximate average of the number of found synteny blocks) of synteny blocks for each comparison (0.05/(2211*500) ≈ 5*e* − 8).

For each synteny block found by PhylDiag, we kept only the genes that had one single exemplar in the two blocks from both species. We counted the number of such pairs of genes, and referred to an LCA reconciliation of the output trees of ProfileNJ to check that most pairs are orthologs (their common ancestor is labeled by a speciation). We discarded the blocks that did not fit this condition. This discards possible block duplications.

For the remaining blocks, and for each couple of uniquely represented genes *a* and *b*, we required that the LCA node *X* of *a* and *b* in the reconciled ProfileNJ tree is not a supported duplication: let *X*_1_ and *X*_2_ be the two children of the node *X* labeled as a duplication (so *X*_1_ and *X*_2_ are in the same species as *X*), the genes *a* and *b* are not kept as putative orthologs if one of the branches *XX*_1_ and *XX*_2_ has a high support (>0.95), and there are two genes, *x*_1_ and *x*_2_, which respectively descend from *X*_1_ and *X*_2_, which are located on the same genome. This discards possible tandem duplications in the block, followed by differential losses of copies.

The output trees from ProfileNJ as well as the filtered pairs of putative orthologs were given as input to ParalogyCorrector, which finds the tree that is as close as possible to the input tree in terms of RF distance, such that in an LCA reconciliation, all pairs of putative orthologs have an LCA node annotated as a speciation.

### Information from ancestral synteny

**Fig 9:**
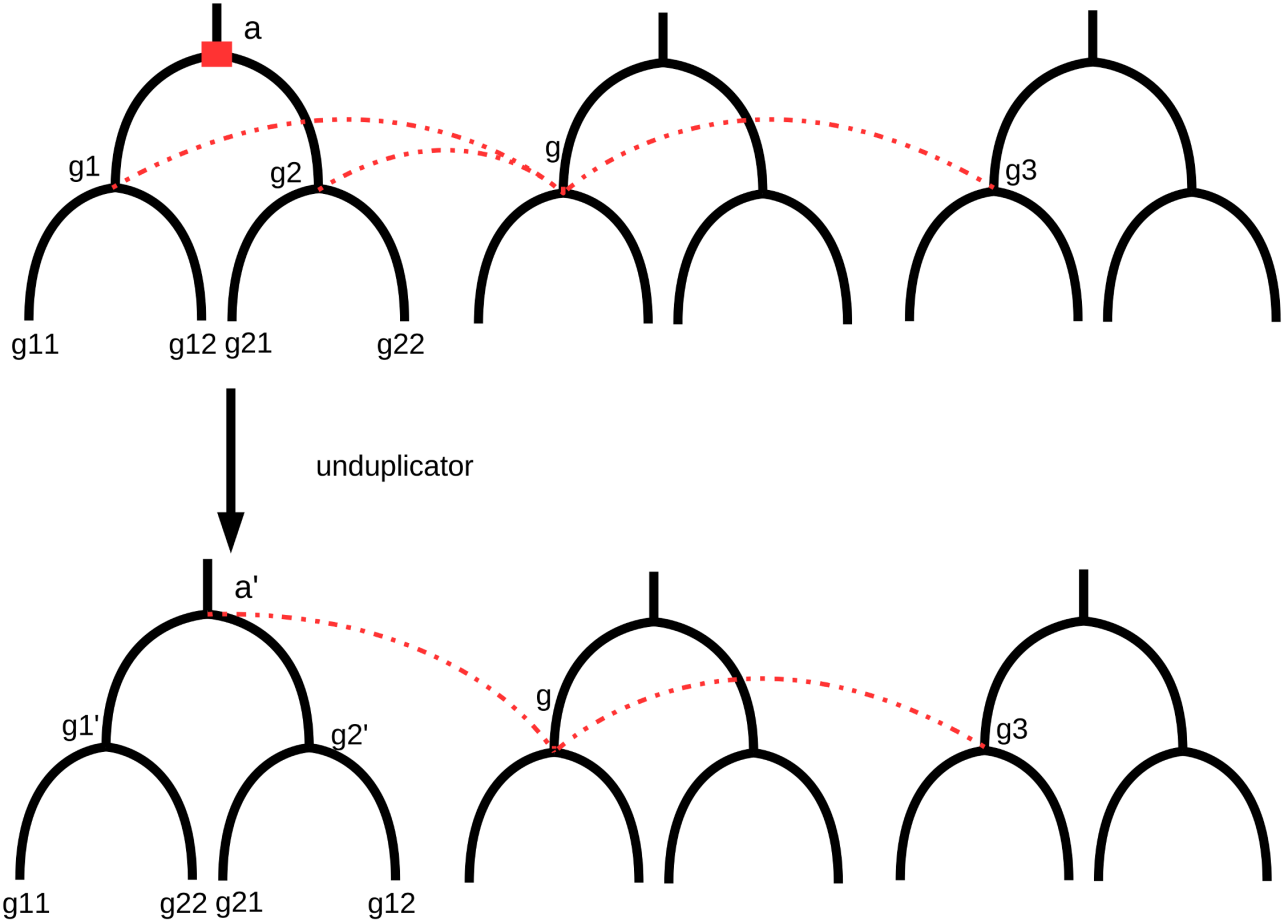
The unduplication principle (figure redrawn from [33]). A non linearity is detected in an ancestral genome (gene *g* has three neighbors). Two of its neighbors *g*_1_ and *g*_2_ are issued from a possibly dubious duplication labeled node. The tree is rearranged so that its root is labeled with a speciation instead of a duplication. In the resulting configuration $g_{1}^{\prime }$ and $g_{2}^{\prime }$ are in two different species, so that *g* can have only one neighbor in this family, and linearity is recovered.

From the results of DeCo on the output gene trees produced by ProfileNJ, we used an “unduplication” principle as in [33] every time we found that an ancestral gene *x* had three neighbors *a*, *b*, *c*, two of them (say *a*, *b*) arising from a duplication node *d* in a single gene tree. In that case, we rearranged the four grand children of *d* so that the clade under *d* has an LCA which is annotated as a speciation in the LCA reconciliation. See an insight into its functioning in Fig 9.

### Likelihood ratio tests

We computed the likelihood of all trees according to the HKY85 model with PhyML on nucleotide alignments. To test the significance of a likelihood difference, we computed the AU tests (Approximately unbiased, [44] with RAxML and Consel [56]. Unless otherwise stated, we use “significantly” better for a tree with a AU value >0.95.

## Supporting Information

S1 File Additional validity and robustness tests of ProfileNJ, followed by a representation of the number of losses along a phylogeny inferred from ProfileNJ trees. Figs A to I are included in S1 File.
(PDF)
