# Supplementary material for: Efficient Gene Tree Correction Guided by Genome Evolution
Source: PLoS One. 2016 Aug 11;11(8):e0159559. doi: 10.1371/journal.pone.0159559 (PMC4981423; doi:10.1371/journal.pone.0159559)
Supplement: S1 File — Figs A to I are included in S1 File. (PDF) [file pone.0159559.s001.pdf]

Efficient gene tree correction guided by genome evolution

Supplementary Data

Emmanuel Noutahi*[1], Magali Semeria*[2],
Manuel Lafond[1], Jonathan Seguin[1],
Bastien Boussau[2], Laurent Gueguen[2],
Nadia El-Mabrouk[1,4], Eric Tannier[2,3,4]

1 - Département d'Informatique (DIRO), Université de Montréal, H3C3J7, Canada;

2 - LBBE, UMR CNRS 5558, Université de Lyon 1, F-69622 Villeurbanne, France;

3 - INRIA Grenoble Rhône-Alpes, F-38334 Montbonnot, France

4 - Corresponding authors mabrouk@iro.umontreal.ca, Eric.Tannier@inria.fr

* - equal contribution

Unsupported branches

Figure A shows the distribution of unsupported branches in PhyML trees.

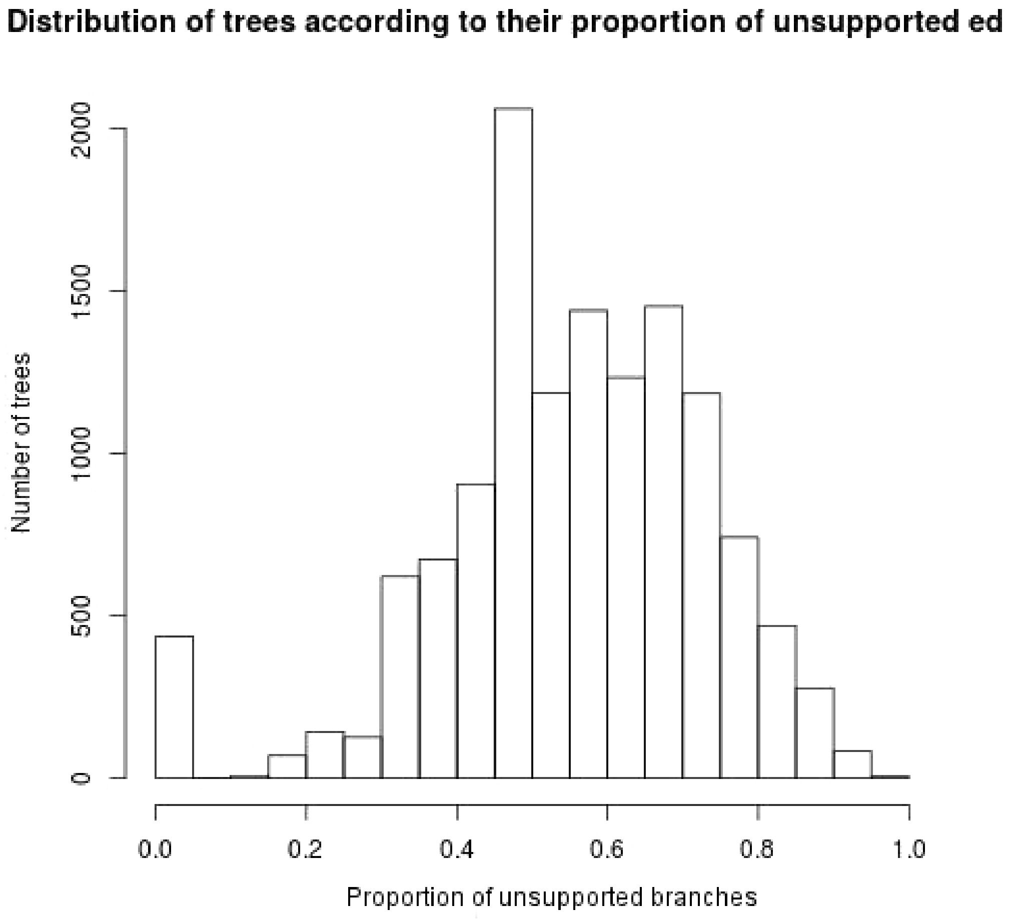

Figure A: Distribution of the number of unsupported (aLRT < 0.95) branches in PhyML trees on the 20529 Ensembl 73 gene families.

RefineTree interface

RefineTree is released with a web interface involving ProfileNJ and ParalogyCorrector. The user is requested to provide a species tree, or alternatively point to the Ensembl species tree, and a gene tree or an Ensembl gene tree ID. The distance matrix used in ProfileNJ can be provided or computed from the nucleotide or amino acids sequences input by the user. Such sequences are also required for ranking solutions by their likelihood using PhyML. A graphical representation of the corrected tree is displayed using the ETE2 Python Framework [1]. An example is given in Figure B.

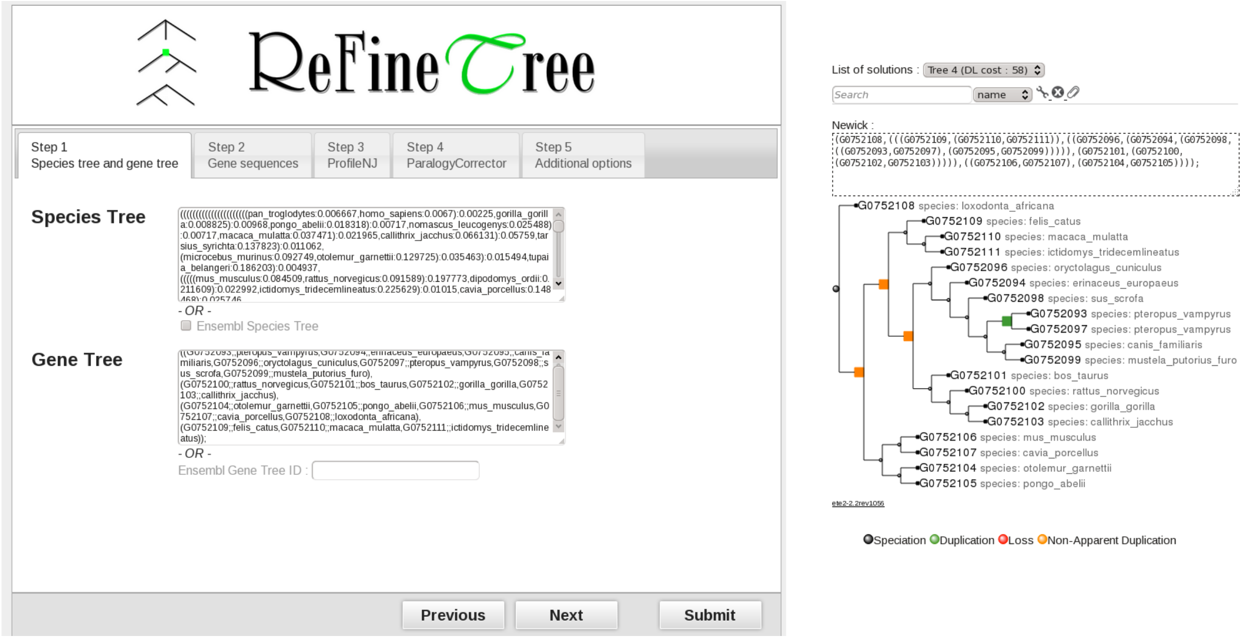

Figure B: RefineTree web interface. The main screen on the left, and an example of an output on the right with speciation and duplication nodes labelled differently.

Simulation

We provide the results of several analyses that show the behavior of ProfileNJ, compared to TreeFix, on simulations.

Topology Accuracy: Accuracy of tree topology is evaluated according to the Robinson-Foulds (RF) distance, i.e. the number of symmetric differences between the clade-sets, of the output tree and the true tree (obtained from simulations).

This difference between a reconciliation-based method versus a pure sequence-based method is probably related to the evolutionary model chosen for simulations, which appears to fit the parsimony criterion for duplications and losses, and thus favour a method based on the reconciliation cost. Indeed, among the simulated data-sets with true estimated DL rates ($1r_D$ - $1r_L$), TreeFix and ProfileNJ are able to recover about 93% of correct topologies. Moreover, a decrease in topology accuracy of both programs for increasing number of evolutionary events is observed (Figure C), TreeFix performing slightly better than ProfileNJ. This is expected as the most parsimonious reconciliation will always lead to the fewest number of events required to explain the data. For small trees with high DL events, ProfileNJ might not therefore be able to recover the correct tree. This artefact is not observed on simulated data-sets with true estimated DL rate.

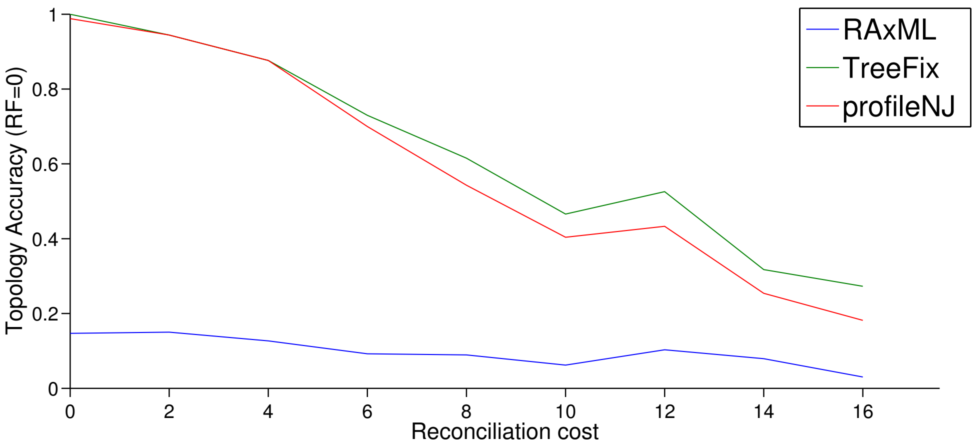

Figure C: Reconstruction accuracy of RAxML, TreeFix and ProfileNJ for increasing cost of reconciliation. Both TreeFix and ProfileNJ accuracy decrease when the number of evolutionary events increase, with TreeFix performing slightly better, but both still outperform RAxML.

Most cases of ProfileNJ errors in tree topologies (non-zero RF distance) are due to duplications that have been predicted lower on the tree. This is a known bias of reconciliation-based reconstruction methods, as lower duplications lead to less loss predictions. An example is given in Figure D.

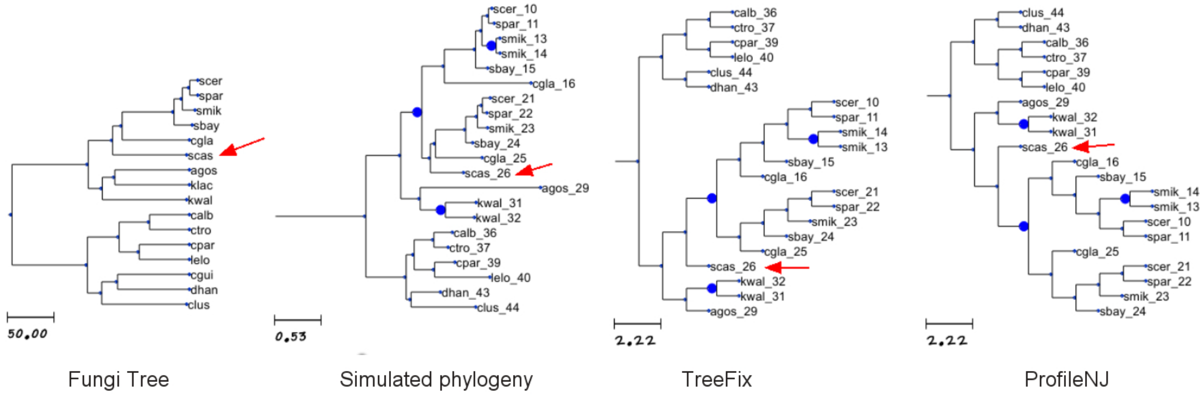

Figure D: An example of erroneously predicted topologies by TreeFix and ProfileNJ. Minimizing losses led both algorithms to place scas_26 above the predicted duplication, while the true (non parsimonious in terms of DL) scenario is a loss in scas.

Duplication and Loss Accuracy: Figure E illustrates accuracy of the duplication, loss and mutation (loss+duplication) costs, measured as the ratio of correct over all predictions, where a correct prediction is an output tree exhibiting the same cost as the true tree.

Again, ProfileNJ and TreeFix exhibit similar results, with ProfileNJ performing slightly better. High accuracy is obtained for all three measures, even though slightly lower for losses. This is due to the tendency of reconciliation-based methods to predict duplications lower in the tree, as illustrated in Figure D. On

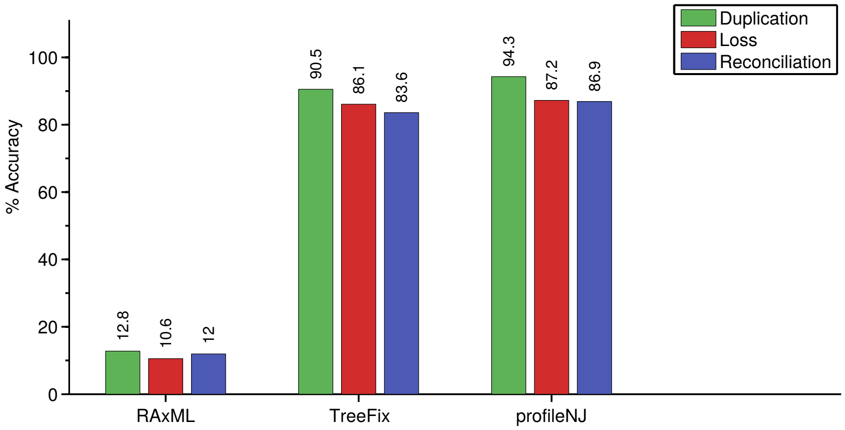

Figure E: Accuracy of duplications, losses and reconciliation cost of TreeFix, ProfileNJ and RaxML trees on ~ 2500 simulated data from the fungi data-set.

the other hand, RAxML trees generally have a higher reconciliation cost (88% of RAxML trees have a reconciliation cost higher than the true cost, against 9.20% for TreeFix and 1.44% for ProfileNJ).

Gene Tree Size effect on Accuracy: To evaluate the scalability of the algorithms to gene trees of different sizes, we subdivided the set of trees into six classes according to their number of leaves: 0-10, 10-20, 20-30, 30-40, 40-50 and 50-60. Figure F shows that performance of all algorithms decrease with increase of tree size. However, ProfileNJ reconciliation cost accuracy is the less affected by the increase in tree size.

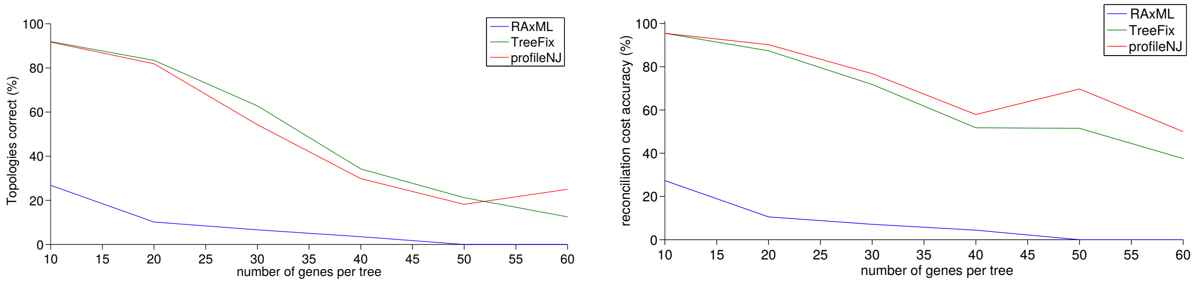

Figure F: Topological accuracy (left diagram) and reconciliation cost accuracy (right diagram) of TreeFix, ProfileNJ and RAxML for increasing size of gene tree from ~ 2500 simulated fungal data-set.

Statistical Support: The AU test (using consel) has been used for evaluating the statistical support of trees according to sequence data. Figure G shows very similar results for the simulated tree and both TreeFix and ProfileNJ output trees, and much better results for the tree output by RAxML. This is not surprising as the RAxML tree is the ML tree. Here, for the large majority of data-sets, the simulated tree is not the ML tree, which invalidates the use of a sequence-based method such as RAxML for tree reconstruction. TreeFix performs better than ProfileNJ as the trees failing the AU test at $\alpha = 0.05$ represents 1.36% of all trees for

TreeFix and 9.165% of all trees for ProfileNJ. This is expected as TreeFix admits a corrected tree only if it is statistically equivalent to the input tree, while ProfileNJ outputs, among the optimal resolutions, the one best fitting the sequences.

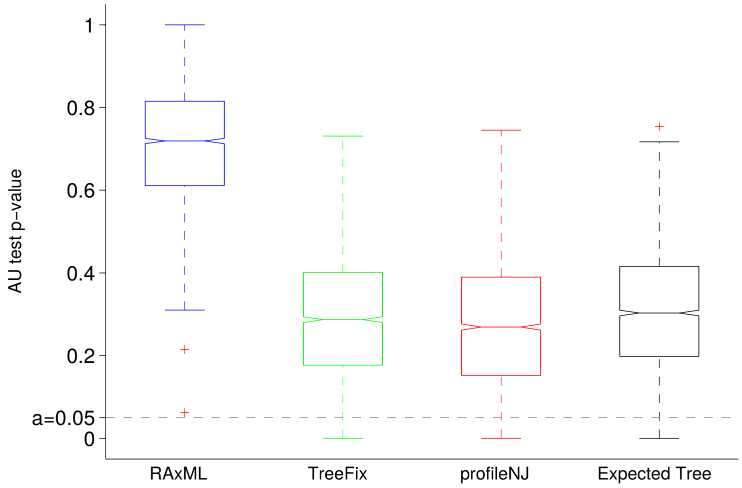

Figure G: Range of the p-value of an AU test using consel, between RAxML, TreeFix, ProfileNJ outputs and the simulated tree.

Robustness to Errors in the Species Tree: Reconstruction methods using information on the species tree are dependant upon the quality of the used tree. To measure this dependancy, we considered an alternative species tree for fungi, obtained by [2] (Figure I). Results on topology accuracy are given in Figure H. By comparing with Figure 2 (from the main text), it clearly appears that accuracy of both ProfileNJ and TreeFix is significantly affected by this erroneous species tree. This drop of accuracy is somewhat lower for ProfileNJ than for TreeFix. Moreover, RAxML outperforms both algorithms in this case. A pure sequence-based method could therefore be more appropriate when there is ambiguity in the species tree. This is a clear limitation of a reconciliation-based reconstruction method. We believe, however, that correcting only branches with low support leads to less dependency on the specie tree if the contraction threshold is cautiously chosen. This is supported by the result of Figure I which show improvement of ProfileNJ tree, on an incorrect specie tree, for lower contraction thresholds.

Modes of evolution in eukaryotes

Figure J gives the number of losses per branch of the phylogeny.

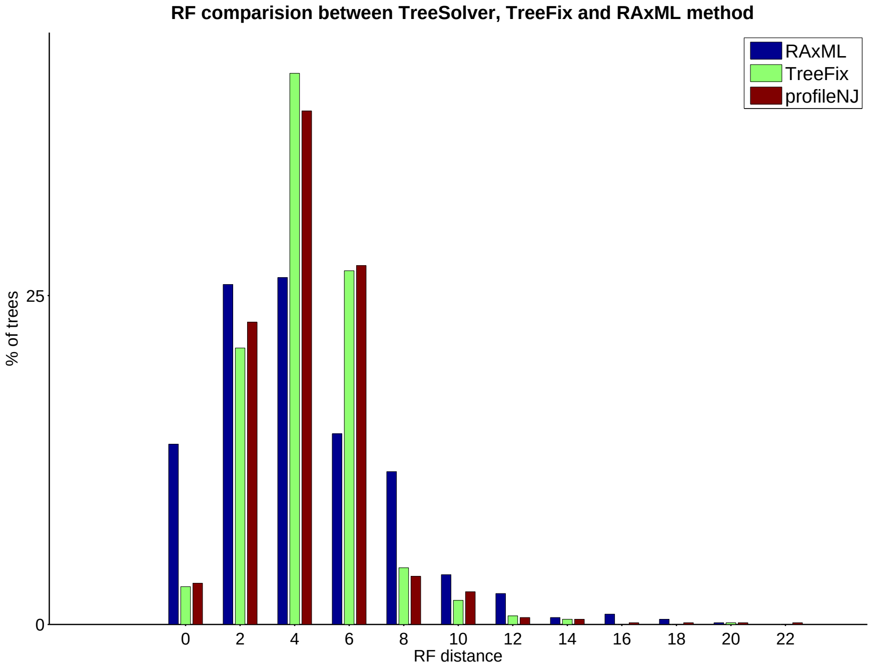

Figure H: Topology accuracy of RAxML, TreeFix and ProfileNJ, mesured by RF distance with the true tree, on $\sim$ 2500 simulated trees from the fungal data-set, using incorrect species tree topologies. TreeFix and ProfileNJ lost their high accuracies while no effect is seen for RAxML as its output is not affected by the species tree.

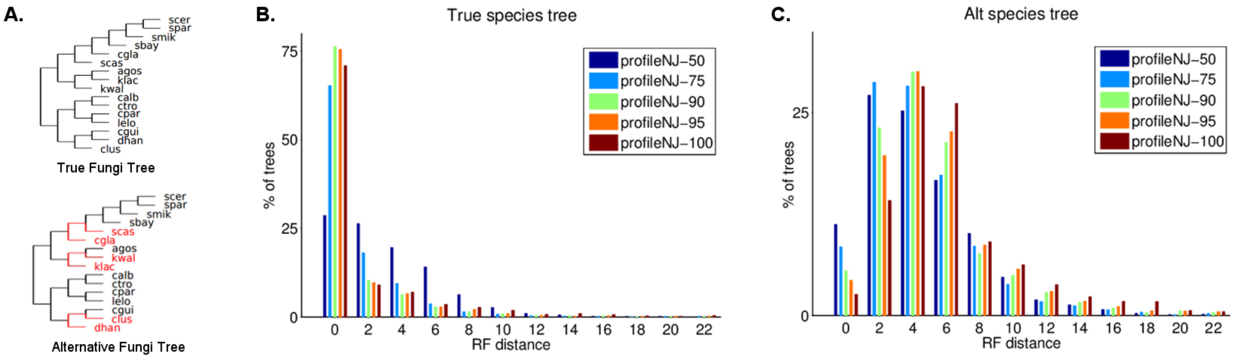

Figure I: Topology accuracy of ProfileNJ for different contraction threshold (α) using the correct and incorrect species tree. **(A)** True and alternative fungi specie trees. **(B)** Topology accuracy of ProfileNJ under the true specie tree. On those simulated data, we obtained the best performance when $\alpha = 90\%$ and $\alpha = 95\%$. Topology accuracy drop for lower values of α as incorrect branches are accepted and the tree space exploration is reduced. **(C)** Topology accuracy of ProfileNJ with an alternative specie tree. Here, lower values of α produce better tree, suggesting that α can be used as a way to control uncertainty in either the branch of the gene tree or the specie tree information.

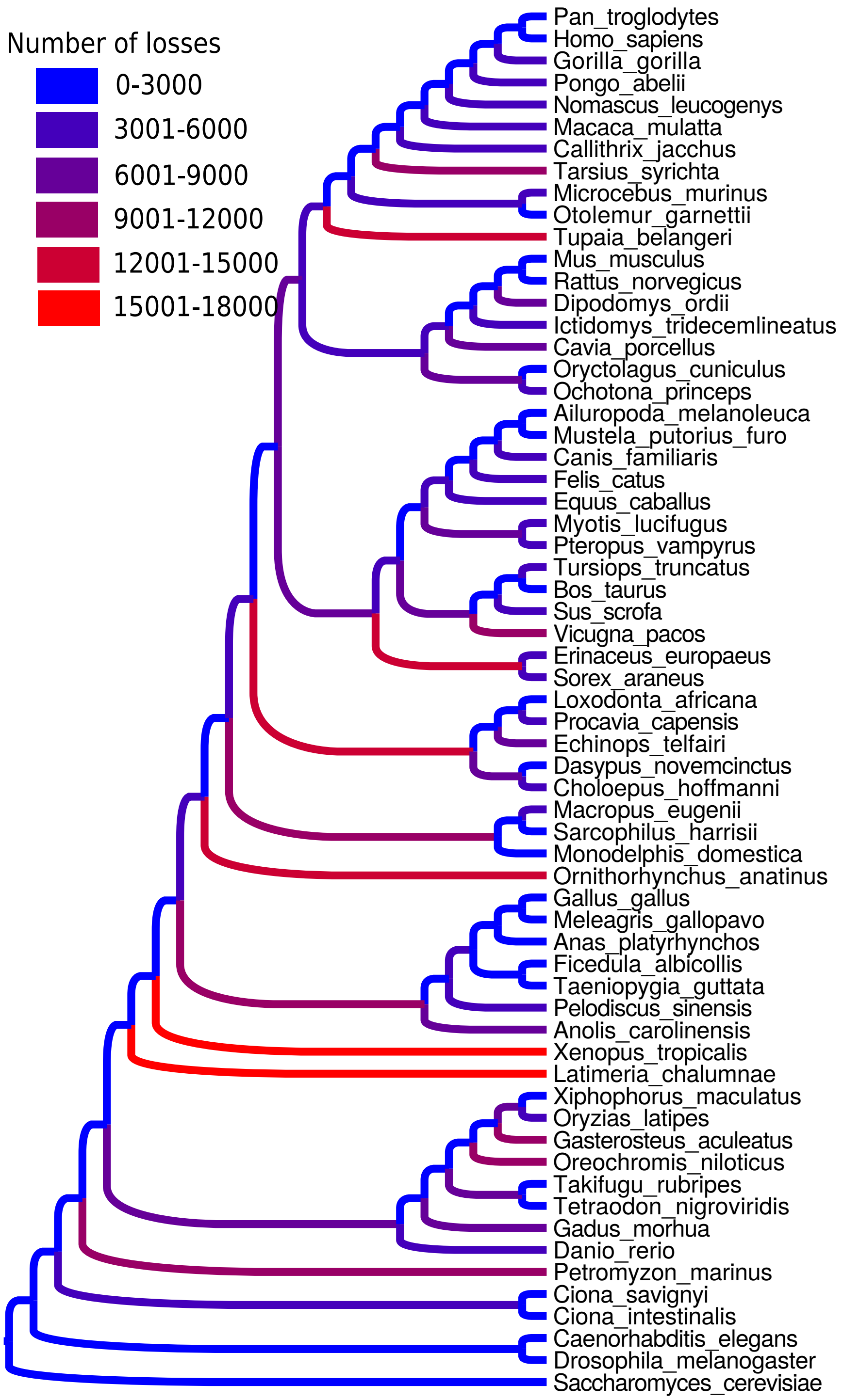

Figure J: Numbers of losses in the eukaryote phylogeny, estimated with ProfileNJ trees from PhyML starting trees on the whole Ensembl Compara database, version 73.

Reference

[1] Huerta-Cepas J, Dopazo J, Gabaldon T. ETE: a python Environment for Tree Exploration. BMC Bioinformatics. 2010;11(1):24. Available from: http://www.biomedcentral.com/1471-2105/11/24.

[2] Wu YC, Rasmussen MD, Bansal MS, Kellis M. TreeFix: Statistically informed gene tree error correction using species trees. Systematic Biology. 2013;62(1):110- 120.
